# Claim Denials for Cancer-Related Next-Generation Sequencing in Medicare

**DOI:** 10.1001/jamanetworkopen.2025.5785

**Published:** 2025-04-18

**Authors:** So-Yeon Kang, Ilina Odouard, Carole Roan Gresenz

**Affiliations:** 1 Department of Health Management and Policy, Georgetown University School of Health, Washington, DC; 2 Department of Health Policy and Management, Johns Hopkins Bloomberg School of Public Health, Baltimore, Maryland; 3 McCourt School of Public Policy, Georgetown University, Washington, DC

## Abstract

**Importance:**

The Centers for Medicare & Medicaid Services implemented a National Coverage Determination (NCD) for genetic testing using next-generation sequencing (NGS) in March 2018 and amended it in January 2020. Little is known about how often NGS insurance claims are denied and what factors are associated with these denials.

**Objective:**

To examine the prevalence of and factors associated with claim denials for NGS testing among Medicare enrollees.

**Design, Setting, and Participants:**

This cohort study used a 20% random sample of Medicare claims data from January 1, 2016, to December 31, 2021. Statistical analysis took place from June to October 2024.

**Main Outcomes and Measures:**

Claim denials were measured based on payment and denial data. Multivariable logistic regression models were estimated with patient and claim characteristics, including testing type, testing site, and time period indicators reflecting the presence and scope of the NCD: no NCD (period 1), first NCD (period 2), and amended NCD (period 3).

**Results:**

The study sample included 29 919 cancer-related NGS claims among 24 443 unique Medicare beneficiaries (51.8% males; 44.7% aged >75 years). The number of cancer-related NGS testing claims increased from 1912 in 2016 to 9177 in 2021. Claim denial occurred among 23.3% of the NGS claims in the sample. The claim denial rate was 16.8% before the NCD, 20.3% after the implementation of the NCD in 2018, and 27.4% after the amended NCD in 2020. Claims for NGS testing were more likely to be denied if they were performed in independent laboratories (odds ratio [OR], 2.76 [95% CI, 2.58-2.95]; *P* < .001) or other nonhospital sites (OR, 2.55 [95% CI, 2.12-3.07]; *P* < .001) vs a hospital or if they were for 50 or more genes vs 50 or fewer genes for solid tumors (OR, 1.32 [95% CI, 1.23-1.43]; *P* < .001). The likelihood of a claim denial was higher in both time periods after the original 2018 NCD compared with before the NCD (period 2: OR, 1.23 [95% CI, 1.10-1.36]; *P* < .001; period 3: OR, 1.64 [95% CI, 1.49-1.80]; *P* < .001). The claim denial likelihood for period 3 was significantly higher than period 2 (OR, 1.34 [95% CI, 1.24-1.44]; *P* < .001).

**Conclusions and Relevance:**

In this cohort study of cancer-related NGS testing claims among Medicare enrollees, denial rates varied by testing type and testing site and increased over time. The findings suggest the continued existence of uncertainty regarding coverage for NGS, despite the NCD. Policy approaches to further reduce uncertainty regarding NGS coverage and raise awareness of potential financial liability warrant consideration.

## Key Points

**Question:**

What is the prevalence of claim denials for next-generation sequencing (NGS) testing among Medicare enrollees?

**Findings:**

In this cohort study that included 29 919 cancer-related NGS claims among 24 443 unique Medicare beneficiaries, 23.3% of cancer-related claims for NGS testing among Medicare enrollees were denied from 2016 to 2021, and the denial rate increased over time. The likelihood of a denial varied by testing type and site.

**Meaning:**

The findings suggest the continued existence of uncertainty regarding the boundaries of coverage for NGS despite the implementation of the National Coverage Determination.

## Teaser

This cohort study examines the prevalence of and factors associated with claim denials for next-generation sequencing testing among Medicare enrollees from 2016 to 2021.

## Introduction

Genomic testing is an increasingly common service that is frequently costly and variably covered by health insurance plans.^1,2^ The complexity of and differences across health plans in coverage standards for genomic testing can generate risk for patients who receive the service, as costs of testing that has already been performed but is not covered by insurance can, in some circumstances, become the patient’s liability.^3^ Unexpected costs of care can have adverse financial consequences for patients and may influence subsequent treatment adherence.^4^ Genomic testing among enrollees in traditional, fee-for-service Medicare has received particular scrutiny because of its rapid growth—with Medicare spending on 4 categories of high-priced genetic testing increasing from $1.2 billion to $1.9 billion between 2020 and 2021—and concerns about excessive use and fraud, both of which can expose patients to the risk of financial liability.^5,6^

In March 2018, the Centers for Medicare & Medicaid Services (CMS) implemented a National Coverage Determination (NCD) for next-generation sequencing (NGS). Next-generation sequencing is an advanced genomic testing technology that enables the simultaneous analysis of multiple tumor genomic markers,^7^ offering more precise information about various tumor mutations to both patients and physicians and other health care professionals.^8^ Under the current provisions of the NCD, US Food and Drug Administration (FDA)–approved NGS is covered nationally for testing both somatic (ie, tumor) mutations and germline (ie, inherited) mutations in a certified laboratory when ordered by a treating physician for patients who have not been previously tested with NGS and who seek further cancer treatment.^9^ The initial NCD in 2018 focused on somatic testing and was limited to patients with recurrent, relapsed, refractory, metastatic, or advanced stages III or IV cancer.^9^ In January 2020, the NCD was extended to germline testing for individuals who have received a diagnosis of or who have a risk factor for breast or ovarian cancer, as well as those who have a clinical indication for germline testing for those cancers.

Although the NCD clarified and universalized coverage standards for NGS testing in these specific circumstances, Medicare Administrative Contractors have discretion over the coverage of diagnostic NGS for non–FDA-approved or non–FDA-cleared tests if patients otherwise meet the criteria for somatic or germline NGS testing. Likewise, since 2020, Medicare Administrative Contractors have discretionary coverage for germline testing for nonovarian or breast cancer and other risk factors for hereditary cancer.^9^ Uncertainty about coverage standards and associated reimbursement challenges can be a barrier to the use of NGS testing.^10^

Prior research has examined changes in NGS use after the NCD implementation, as well as the association between NGS use and sociodemographic characteristics, such as race and ethnicity and socioeconomic status.^11,12,13^ These studies document an upward trend in NGS testing after the NCD implementation but found that implementation of the NCD did not alleviate racial and ethnic disparities in NGS use.^11,13^ Furthermore, a study on metastatic prostate and urothelial cancers found that, despite the overall increase in NGS testing rates, most patients still do not undergo testing, highlighting potential disparities in access to or use of NGS testing.^12^ Other research assessing insurance coverage of tumor sequencing suggests the NCD may influence commercial payers’ coverage decisions.^14^ However, little is known about how often insurance claims for NGS testing are denied, what factors may be associated with the likelihood of a claim denial, and the potential provider (eg, hospitals, physician offices) or patient financial liability that is associated with claim denials for NGS testing.

In this study, we use adjudicated claims from 2016 to 2021 to analyze claim denials for cancer-related NGS testing among traditional, fee-for-service Medicare enrollees. We describe NGS claim denial rates and examine factors associated with the likelihood of a claim denial. We also describe the charge amount for denied claims, which we use as a proxy for the potential financial liability associated with payment denial, which can fall to either providers or patients.^4^

## Methods

### Data and Study Sample

For this cohort study, we used Medicare fee-for-service carrier and outpatient claims from January 1, 2016, to December 31, 2021. These data include a 20% nationally representative, random sample of all Medicare beneficiaries enrolled in Medicare Parts A and B. The dataset contains fully adjudicated, final action claims in which all adjustments to earlier claims have been resolved. It provides the Healthcare Common Procedure Coding System (HCPCS) code, the charge amount for the service of the claim line, the Medicare payment amount, and the claim denial code for the line claim.^4,15^ We identified claims billed for NGS testing using HCPCS codes 81445 (solid tumors, 5-50 genes), 81450 (hematologic malignant neoplasms, 5-50 genes), and 81455 (solid or hematologic malignant neoplasms when >50 genes are analyzed).^16^ These are specified in Medicare’s coding guidelines for NGS and have been used in prior research and publicly available coding and reimbursement policies on NGS.^17,18,19,20^ We excluded claims for which the principal diagnosis code was a noncancer indication. Claims were also excluded if they did not include an HCPCS code or a unique enrollee identifier or if they had a missing value in the charge amount. We supplemented the data with the Medicare Beneficiary Summary File Base to identify patient characteristics (sex, age, and race and ethnicity) for the NGS claims. Sex, age, and race and ethnicity were sourced from Medicare enrollment information in the CMS Common Medicare Environment for the NGS claims to control for potential confounding effects of these patient characteristics on the association between claim denials and other variables. This study was exempt from review by the institutional review board at Georgetown University owing to the use of deidentified data without constituting human participants, and informed consent was not sought. The study followed the Strengthening the Reporting of Observational Studies in Epidemiology (STROBE) reporting guideline for observational studies.

Claims with the claim denial code value of “denied” were classified as claims rejected for payment as a final determination. We checked the claim denial code against the Medicare payment amount to confirm the coding of the denial; all denied claims had a zero payment amount. We also obtained the reason for claim denial, which can be based on medical grounds, such as medical necessity or diagnosis, or reflect administrative issues, such as incomplete or incorrect information or redundant submissions. We used the charge amount (adjusted to 2021 US dollars) as a proxy for the maximum potential health care professional or patient financial liability for a denied NGS testing claim. We were not able to ascertain whether the patient or health care professional was liable for payment. For patients, the amount represents an upper bound on potential liability because supplemental insurance may cover some of the cost, and some testing providers may offer price discounts if insurance fails to pay.^4^

We defined 3 time periods based on the presence and scope of the NCD: period 1 was from January 2016 to February 2018 before the initial NCD was in place; period 2 was the time period from the March 2018 NCD for somatic testing through December 2019; and period 3 was the time period after the amended NCD extending coverage for germline testing in January 2020. We created indicators for the site of testing (hospital outpatient center, independent laboratory, and other nonhospital site) and type of testing (solid tumor, 5-50 genes; hematologic malignant neoplasm, 5-50 genes; and solid or hematologic malignant neoplasm, >50 genes). Patient-level variables included age, sex, age group, race and ethnicity, and geographic location (state of residence), which previous research suggests may be associated with other types of claim denials.^21^

### Statistical Analysis

Statistical analysis took place from June to October 2024. We assessed differences in sample characteristics for paid vs denied NGS claims using Pearson χ^2^ tests. We summarized claim denial rates by NCD implementation status (periods 1-3), HCPCS code, and testing site. We analyzed the likelihood of a claim denial using multivariable logistic regression applied to claims-level data. The dependent variable was a binary indicator for whether a claim was denied, and we controlled for patient (age, sex, race and ethnicity, and state of residence) and claim (type of testing, site of testing, and first vs subsequent NGS testing) characteristics. In our main specification, we included indicator variables for whether NGS testing was performed when the first or second version of the NCD was in effect (dummy variables for periods 2 and 3). We clustered the SEs by the patient. We conducted sensitivity analyses in which we varied the covariate mix and in which we included only the first observed NGS claim.

The threshold for statistical significance was α = .05 using 2-sided tests. All analyses were conducted in Stata, version 17 (StataCorp LLC).

## Results

### Characteristics of Paid vs Denied Claims for NGS Testing

The study sample included 29 919 claims for cancer-related NGS testing among 24 443 unique Medicare beneficiaries (51.8% male and 48.2% female; 44.7% aged >75 years; and 2.1% Asian beneficiaries, 6.6% Black beneficiaries, 1.2% Hispanic or Latinx beneficiaries, 4.5% North American Native, unknown, or other beneficiaries, and 85.6% White beneficiaries) from 2016 to 2021 (Table 1). Among the cancer-related NGS testing claims, 35.9% were for testing of solid tumors (5-50 genes), 36.8% for hematologic malignant neoplasms (5-50 genes), and 27.3% for testing of solid or hematologic malignant neoplasms (>50 genes). Most claims were for NGS tests conducted at hospital outpatient sites (52.3%), while 45.1% of claims were for testing conducted in independent laboratories.

**Table 1.** Characteristics of Paid vs Denied Claims for NGS Testing Among Medicare Fee-for-Service Beneficiaries, 2016-2021^a^

Characteristic	No. (%)			*P* value
	All claims	Paid claims	Denied claims	
Unique claims	29 919 (100)	22 952 (76.7)	6967 (23.3)	NA
NGS tests per patient, mean (SD), No.	1.2 (0.7)	1.2 (0.7)	1.2 (0.7)	.03
Sequence of NGS testing for the patient, mean (SD)	1.3 (0.8)	1.3 (0.8)	1.3 (0.9)	.25
HCPCS code				
81445 (Solid tumors, 5-50 genes)	10 744 (35.9)	7898 (34.4)	2846 (40.9)	<.001
81450 (Hematologic malignant neoplasms, 5-50 genes)	11 011 (36.8)	9497 (41.4)	1514 (21.7)	
81455 (Solid or hematologic malignant neoplasms, >50 genes)	8164 (27.3)	5557 (24.2)	2607 (37.4)	
Site of testing				
Hospital outpatient centers	15 641 (52.3)	13 181 (57.4)	2460 (35.3)	<.001
Independent laboratories	13 499 (45.1)	9306 (40.6)	4193 (60.2)	
Other nonhospital site^b^	779 (2.6)	465 (2.0)	314 (4.5)	
Cancer type				
Solid tumors	19 077 (63.8)	13 666 (59.5)	5411 (77.7)	<.001
Hematologic malignant neoplasms	10 842 (36.2)	9286 (40.5)	1556 (22.3)	
Patient characteristics				
Sex				
Male	15 511 (51.8)	11 921 (51.9)	3590 (51.5)	.55
Female	14 408 (48.2)	11 031 (48.1)	3377 (48.5)	
Age, y				
<65	2158 (7.2)	1633 (7.1)	525 (7.5)	.01
65-69	6438 (21.5)	4853 (21.1)	1585 (22.8)	
70-74	7947 (26.6)	6118 (26.7)	1829 (26.3)	
>75	13 376 (44.7)	10 348 (45.1)	3028 (43.5)	
Race and ethnicity				
Asian	625 (2.1)	475 (2.1)	150 (2.2)	.049
Black	1979 (6.6)	1490 (6.5)	489 (7.0)	
Hispanic or Latinx	351 (1.2)	252 (1.1)	99 (1.4)	
North American Native, unknown, or other^c^	1350 (4.5)	1014 (4.4)	336 (4.9)	
White	25 614 (85.6)	19 721 (85.9)	5893 (84.6)	

Abbreviations: HCPCS, Healthcare Common Procedure Coding System; NA, not applicable; NGS, next-generation sequencing.

^a^ Source: Medicare fee-for-service claims (20% random sample). The period before the National Coverage Determination (NCD) for NGS testing was between January 1, 2016, and February 28, 2018. The period after the NCD for NGS testing was between March 1, 2018, and December 31, 2021.

^b^ Other nonhospital site includes physician offices.

^c^ Other and unknown race and ethnicity are the race categories specified in the Medicare Beneficiary Summary File.

Claim denial occurred among 23.3% of the NGS claims in the sample (Table 1). The percentage of cancer-related NGS testing claims denied increased from 16.8% prior to the NCD to 20.3% after its 2018 implementation and to 27.4% after the 2020 NCD amendment. The mean (SD) number of NGS tests per beneficiary was 1.2 (0.7). The distribution of claims by NGS type and site of testing differed between paid and denied claims. Among the NGS test types, HCPCS code 81445 (solid tumors, 5-50 genes) accounted for the largest share of denied claims (40.9%) compared with HCPCS code 81450 (hematologic malignant neoplasms, 5-50 genes; 21.7%) and HCPSC code 81455 (solid or hematologic malignant neoplasms, >50 genes; 37.4%; *P* < .001). Claims from independent laboratories (vs hospitals) accounted for a higher proportion of denied claims than paid claims (60.2% vs 40.6%; *P* < .001). The reverse was true for hospital claims; 35.3% of denied claims came from hospitals, while 57.4% of paid claims came from hospitals (*P* < .001).

### Claim Denial Rates and Potential Health Care Professional or Patient Financial Liability for Denied Claims

The number of cancer-related NGS testing claims increased from 1912 in 2016 to 9177 in 2021. During the same period, the claim denial rate increased from 18.4% in 2016 to 33.3% in 2021, with the biggest year-to-year increase in the denial rate between 2020 and 2021 (Figure). The claim denial rate for NGS testing performed by independent laboratories was 27.8% in period 1, 22.6% in period 2, and 37.3% in period 3 (Table 2). The claim denial rate for claims with HCPCS code 81455 (testing for >50 genes) increased from 16.3% in period 1 to 18.6% in period 2 to 40.3% in period 3. The median charge amount among denied NGS claims, which represents the upper limit of health care professional or patient liability, was $3800 (IQR, $2650-$3979) (eTable 1 in Supplement 1).

**Figure.**
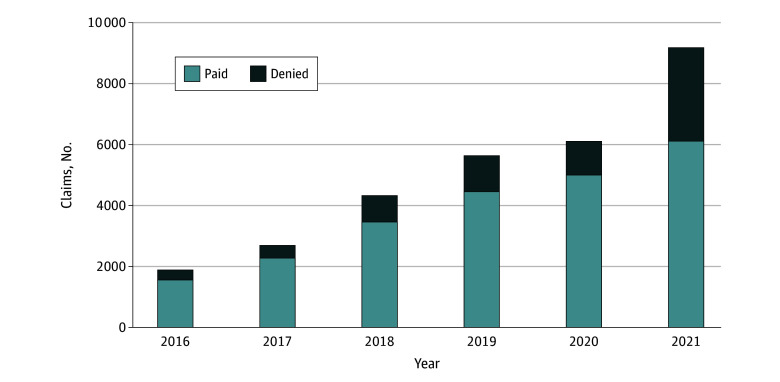
Next-Generation Sequencing (NGS) Claim Denials Among Medicare Beneficiaries, 2016-2021 Source: 20% random sample of Medicare fee-for-service claims for NGS testing with cancer diagnosis.

**Table 2.** NGS Claim Denial Rates by Study Periods, Testing Type, and Testing Site^a^

Variable	Claim denial rate (denied claims/all claims), No./total No. (%)			
	Full period	Period 1 (January 2016 to February 2018)	Period 2 (March 2018 to December 2019)	Period 3 (January 2020 to December 2021)
All claims	6967/29 919 (23.3)	872/5180 (16.8)	1961/9653 (20.3)	4088/14914 (27.4)
HCPCS code				
81445 (Solid tumors, 5-50 genes)	2846/10 744 (26.5)	457/2009 (22.8)	1119/4334 (25.8)	1270/4401 (28.9)
81450 (Hematologic malignant neoplasms, 5-50 genes)	1514/11 011 (13.8)	236/1959 (12.1)	543/3713 (14.6)	735/5339 (13.8)
81455 (Solid or hematologic malignant neoplasms, >50 genes)	2607/8164 (31.9)	225/1384 (16.3)	299/1606 (18.6)	2083/5174 (40.3)
Site of testing				
Hospital outpatient centers	2460/15 641 (15.7)	263/3153 (8.3)	876/5062 (17.3)	1321/7426 (17.8)
Independent laboratories	4193/13 499 (31.1)	579/2081 (27.8)	992/4389 (22.6)	2622/7029 (37.3)
Other nonhospital site	314/779 (40.3)	76/118 (64.4)	93/202 (46.0)	145/459 (31.6)

Abbreviations: HCPCS, Healthcare Common Procedure Coding System; NGS, next-generation sequencing.

^a^ Source: Medicare fee-for-service claims (20% random sample). The period before the National Coverage Determination (NCD) for NGS testing was between January 1, 2016, and February 28, 2018. The period after the NCD for NGS testing was between March 1, 2018, and December 31, 2021.

### Factors Associated With Claim Denials for NGS Testing

Table 3 shows the estimates from the multivariable regression analysis of the likelihood of denial among NGS claims. When controlling for patient and testing service characteristics of NGS use as well as state fixed effects, the likelihood of NGS claim denial was higher for period 2 under the 2018 NCD for somatic testing (odds ratio [OR], 1.23 [95% CI, 1.10-1.36]; *P* < .001) and period 3 under the 2020 NCD extension for germline testing (OR, 1.64 [95% CI, 1.49-1.80]; *P* < .001). The difference between period 2 and period 3 was significant (OR, 1.34 [95% CI, 1.24-1.44]; *P* < .001 for period 3 vs period 2). The HCPCS code 81450 (hematologic malignant neoplasms for ≤50 genes) was less likely to be denied compared with HCPCS code 81445 (solid tumors for ≤50 genes) (OR, 0.41 [95% CI, 0.37-0.44]; *P* < .001). The non–NCD-covered code HCPCS 81455 (any cancer for ≥51 genes) was also more likely to be denied compared with HCPCS code 81445 (OR, 1.32 [95% CI, 1.23-1.43]; *P* < .001). Claims were more likely to be denied for NGS performed at independent laboratories (OR, 2.76 [95% CI, 2.58-2.95]; *P* < .001) and other nonhospital sites (OR, 2.55 [95% CI, 2.12-3.07]; *P* < .001) compared with claims from hospitals. The directions and magnitudes of these estimates remain consistent across different model specifications, including the regression model with cancer subtypes (model 2), subgroup analysis focusing on older adults (model 3) and the first NGS claims (model 4), and the regression model focusing on the 2018 NCD implementation (eTable 2 in Supplement 1).

**Table 3.** Adjusted Association Between NGS Claim Denial and Variables of Interest^a^

Variable	Likelihood of claim denial, OR (95% CI)
Time period	
Period 1 (2016 to February 2018)	1 [Reference]
Period 2 (March 2018 to December 2019)	1.23 (1.10-1.36)^b^
Period 3 (January 2020 to December 2021)	1.64 (1.49-1.80)^b^
Not the beneficiary’s first NGS use (reference: first NGS use)	1.36 (1.25-1.47)^b^
HCPCS code	
81445 (Solid tumors, 5-50 genes)	1 [Reference]
81450 (Hematologic malignant neoplasms, 5-50 genes)	0.41 (0.37-0.44)^b^
81455 (Solid or hematologic malignant neoplasms, >50 genes)	1.32 (1.23-1.43)^b^
Site of testing	
Hospital	1 [Reference]
Independent laboratory	2.76 (2.58-2.95)^b^
Other nonhospital site	2.55 (2.12-3.07)^b^
Sex	
Male	1 [Reference]
Female	0.94 (0.89-1.01)
Age, y	
<65	0.96 (0.84-1.10)
65-69	1 [Reference]
70-74	0.91 (0.83-1.00)^c^
75-80	0.88 (0.81-0.96)^d^
Race and ethnicity	
Asian	0.84 (0.68-1.04)
Black	1.04 (0.91-1.18)
Hispanic	1.29 (0.96-1.74)
North American Native, unknown, or other^e^	1.05 (0.90-1.22)
White	1 [Reference]

Abbreviations: HCPCS, Healthcare Common Procedure Coding System; NGS, next-generation sequencing; OR, odds ratio.

^a^ Source: 20% random sample of Medicare fee-for-service claims. State fixed effects were included in the model but not shown (N = 29 919).

^b^ *P* < .001.

^c^ *P* < .05.

^d^ *P* < .01.

^e^ Other and unknown race and ethnicity are the race categories specified in the Medicare Beneficiary Summary File.

### Reasons for NGS Claim Denials

For noninstitutional claims, although all claims had a value for the reason for denial, a large share (43.0%) were coded as “other” and thus provide limited meaningful data (eTable 3 in Supplement 1). For hospital claims, 84.3% had some information on the reason for the denial, although practical distinctions between categories are unclear. The most common reason for denial was “diagnosis” (67.3%), followed by noncovered services not deemed a “medical necessity” by the payer (18.1%) and noncovered charges (5.1%). Administrative errors, such as incorrect processing information or duplicate line items, accounted for a small share of the hospital claim denials. The percentage of claims falling into this category was relatively constant over time. Before the NCD, a lack of medical necessity (66.1%) was the primary reason for claim denials of hospital NGS claims (eFigure in Supplement 1). After the NCD, the primary reason shifted to denial based on the diagnosis (77.2%). However, it is unclear how these categories are used and whether both are indicative of a determination of medical necessity.

## Discussion

During the study period, nearly one-fourth of cancer-related NGS testing claims among Medicare beneficiaries were denied by Medicare for payment. The use of NGS testing increased substantially over the study time period, and the percentage of cancer-related NGS testing claims denied increased from 16.8% prior to the NCD to 20.3% after its 2018 implementation and to 27.4% after the 2020 NCD amendment.

Although we lack full information on the reason for payment denial, available information on the reason for claim denials among hospital-based claims shows no increase in claim denial rates for administrative reasons. This finding suggests that the increase in denial rates may center around issues of medical necessity despite the implementation and amendment of the NCD. However, the NCD applies only to a narrow set of circumstances, with Medicare Administrative Contractors having discretion in other cases. Coverage standards across Medicare Administrative Contractors may not always be consistent, which could result in uncertainty.^22^ Some claim denials could reflect a lag between clinical treatment guidelines and coverage guidelines.^5,6^ In addition, previous research has found limited or no associations of NCDs in other settings with health care professional behavior.^23^

Claim denials for NGS testing have the potential to adversely affect beneficiaries.^24^ Although health care professionals are often responsible for the costs of services that they order and that are denied payment by Medicare, health care professionals can shift liability for the costs of services that might not be considered medically necessary to patients through Advanced Beneficiary Notices. These notices alert patients that the costs of a service may not be covered by Medicare and require patients to opt in to receive the service with this knowledge. Little information is available regarding how often these notices are used for NGS testing and the consequences for the distribution of financial liability. Liability is especially important in the context of NGS because of the substantial costs associated with such testing, which for patients can have both adverse financial consequences and affect adherence to future treatment.^4,25,26^

Determining ways to limit the use of NGS testing that does not meet coverage guidelines for medical necessity is important for avoiding adverse financial consequences for older adults as well as health care professionals. Further consideration of proposed policy solutions, such as creating a registry of coverage standards to help reduce uncertainty among health care professionals, along with decision aids for physicians to help determine the medical necessity of NGS testing, appears warranted.^10,27,28^

Despite the substantial increase in public and private investment in precision medicine, evidence on insurance coverage and the financial aspects of the innovation has been scarce, presenting a knowledge gap for prescribing physicians, health systems, and policymakers. Enhanced knowledge of the facilitators and barriers to the evidence-based use of genetic testing is a cornerstone in accelerating the development and diffusion of precision medicine.

### Limitations

This study has several limitations. First, we relied on administrative claims data and did not have clinical information on, for example, cancer stage or purpose of testing (eg, somatic vs germline testing), which might have helped to identify NGS use that falls outside coverage guidelines. Second, while we used the charge amount to measure the upper limit of potential health care professional or patient liability, we do not know where financial liability ultimately falls. For patients, the charge amount may exceed actual out-of-pocket payments because of health care professional discounts and supplemental coverage. In addition, the time period of our data, both before and after the implementation of the NCD, is limited. The time span of our data relative to the implementation of each of the NCDs is insufficient for a more formal time series analysis to support causal inference, so our findings should be interpreted with caution. The results of the regression analysis provide the association between the implementation phase of the NCD versions and claim denial, controlling for patient and testing service characteristics of NGS use, but do not provide an estimate of the association of the NCD with the probability of a claim denial. More research with a longer time frame is warranted. Last, this study’s findings may not be generalizable to non-Medicare patients and Medicare Advantage beneficiaries, which presents future research areas.

## Conclusions

In this cohort study of cancer-related NGS testing claims among Medicare enrollees, we found that denial rates varied by testing type and testing site and have increased over time. Our study is not designed to attribute the changes over time to the NCD; rather, our findings suggest the continued existence of uncertainty regarding the boundaries of coverage for NGS despite the NCD. Policy approaches to further reduce uncertainty regarding NGS coverage and raise awareness of potential financial liability warrant consideration.

## Notes

Supplement 1. **eTable 1.** NGS Claim Charge Rates by Testing Type and Testing Site **eTable 2.** Sensitivity Analysis: Adjusted Association Between NGS Claim Denial and Variables of Interest **eTable 3.** Reasons for Claim Denial by Testing Site **eFigure.** Trends in Reasons for Claim Denials for NGS Testing

Supplement 2.
Data Sharing Statement

## References

[1] Phillips KA, Deverka PA, Trosman JR, . Payer coverage policies for multigene tests. Nat Biotechnol. 2017;35(7):614-617. doi:10.1038/nbt.3912 28700544 PMC5553867

[2] Ferreira-Gonzalez A, Ko G, Fusco N, . Barriers and facilitators to next-generation sequencing use in United States oncology settings: a systematic review. Future Oncol. 2024;20(35):2765-2777. doi:10.1080/14796694.2024.2390821 39316553 PMC11572137

[3] Stephenson J. Medical debt burdens millions of US adults. JAMA Health Forum. 2022;3(3):e220910. doi:10.1001/jamahealthforum.2022.0910 36218883

[4] Bai G, Anderson GF. Variation in the ratio of physician charges to Medicare payments by specialty and region. JAMA. 2017;317(3):315-318. doi:10.1001/jama.2016.16230 28114540

[5] Data brief: Medicare Part B spending on lab tests increased in 2021, driven by higher volume of COVID-19 tests, genetic tests, and chemistry tests. U.S. Department of Health and Human Services, Office of Inspector General. Published December 2022. Accessed January 31, 2025. https://oig.hhs.gov/oei/reports/OEI-09-22-00400.pdf

[6] CMS’s oversight of Medicare payments for the highest paid molecular pathology genetic test was not adequate to reduce the risk of up to $888 million in improper payments. U.S. Department of Health and Human Services, Office of Inspector General. Published June 21, 2023. Accessed January 31, 2025. https://oig.hhs.gov/oas/reports/region9/92203010.asp

[7] Qin D. Next-generation sequencing and its clinical application. Cancer Biol Med. 2019;16(1):4-10. doi:10.20892/j.issn.2095-3941.2018.0055 31119042 PMC6528456

[8] Next generation sequencing (NGS) for Medicare beneficiaries with advanced cancer. Centers for Medicare & Medicaid Services. Accessed January 31, 2025. https://www.cms.gov/medicare-coverage-database/view/ncacal-tracking-sheet.aspx?NCAId=296

[9] National coverage determination (NCD): next generation sequencing (NGS). Centers for Medicare & Medicaid Services. Published 2020. Accessed January 31, 2025. https://www.cms.gov/medicare-coverage-database/details/ncd-details.aspx?NCDId=372

[10] Ferreira-Gonzalez A, Hocum B, Ko G, Shuvo S, Appukkuttan S, Babajanyan S. Next-generation sequencing trends among adult patients with select advanced tumor types: a real-world evidence evaluation. J Mol Diagn. 2024;26(4):292-303. doi:10.1016/j.jmoldx.2024.01.005 38296192

[11] Sheinson DM, Wong WB, Meyer CS, . Trends in use of next-generation sequencing in patients with solid tumors by race and ethnicity after implementation of the Medicare national coverage determination. JAMA Netw Open. 2021;4(12):e2138219. doi:10.1001/jamanetworkopen.2021.38219 34882180 PMC8662372

[12] Hage Chehade C, Jo Y, Gebrael G, . Trends and disparities in next-generation sequencing in metastatic prostate and urothelial cancers. JAMA Netw Open. 2024;7(7):e2423186. doi:10.1001/jamanetworkopen.2024.23186 39023888 PMC11258596

[13] Sheinson DM, Wong WB, Flores C, Ogale S, Gross CP. Association between Medicare’s national coverage determination and utilization of next-generation sequencing. JCO Oncol Pract. 2021;17(11):e1774-e1784. doi:10.1200/OP.20.01023 34043456 PMC8600504

[14] Lin GA, Trosman JR, Douglas MP, . Influence of payer coverage and out-of-pocket costs on ordering of NGS panel tests for hereditary cancer in diverse settings. J Genet Couns. 2022;31(1):130-139. doi:10.1002/jgc4.1459 34231930 PMC8893352

[15] Consumer Price Index (CPI) databases: all urban consumers (current series). US Bureau of Labor Statistics. Accessed January 31, 2025. https://www.bls.gov/cpi/data.htm

[16] Billing and coding: genetic testing for oncology. Centers for Medicare & Medicaid Services. Accessed January 31, 2025. https://www.cms.gov/medicare-coverage-database/view/article.aspx?articleId=59125&ver=26

[17] Billing and coding: MolDX: targeted and comprehensive genomic profile next generation sequencing testing in cancer. Centers for Medicare & Medicaid Services. Accessed January 31, 2025. https://www.cms.gov/medicare-coverage-database/view/article.aspx?articleid=55197&ver=31&

[18] Smart D, Moore W, Fosvig SM, Bloom KJ. A preliminary review of adoption of new HCPCS codes for NGS in CMS claims. J Clin Oncol. 2022;40(16)(suppl):e13522. doi:10.1200/JCO.2022.40.16_suppl.e13522

[19] Coding/reimbursement policy—next generation sequencing (NGS) genetic testing. University of Utah Health Plans. Published July 28, 2021. Accessed January 31, 2025. https://doc.uhealthplan.utah.edu/medicalpolicy/reimb-007.pdf

[20] 81445, 81450, 81455 (Genomic sequencing procedures and other molecular multianalyte assays). Maryland Department of Health. Accessed January 31, 2025. https://health.maryland.gov/mmcp/Documents/Targeted%20Genomic%20Sequencing%20Clinical%20Criteria.pdf

[21] Hoagland A, Yu O, Horný M. Social determinants of health and insurance claim denials for preventive care. JAMA Netw Open. 2024;7(9):e2433316. doi:10.1001/jamanetworkopen.2024.33316 39292461 PMC11411384

[22] Wilk AS, Hirth RA, Zhang W, . Persistent variation in Medicare payment authorization for home hemodialysis treatments. Health Serv Res. 2018;53(2):649-670. doi:10.1111/1475-6773.12650 28105639 PMC5867144

[23] Foote SB, Town RJ. Implementing evidence-based medicine through Medicare coverage decisions. Health Aff (Millwood). 2007;26(6):1634-1642. doi:10.1377/hlthaff.26.6.1634 17978383

[24] Studdert DM, Gresenz CR. Enrollee appeals of preservice coverage denials at 2 health maintenance organizations. JAMA. 2003;289(7):864-870. doi:10.1001/jama.289.7.864 12588270

[25] Cottrill A, Cubanski J, Neumann T, Smith K. Income and assets of Medicare beneficiaries in 2023. KFF. Published February 5, 2024. Accessed January 31, 2025. https://www.kff.org/medicare/issue-brief/income-and-assets-of-medicare-beneficiaries-in-2023/

[26] Gronbeck C, Feng H. Variation in ratios of submitted charges to allowed Medicare payment for dermatologic services. JAMA Dermatol. 2022;158(8):958-960. doi:10.1001/jamadermatol.2022.2214 35767266 PMC9244768

[27] CMS Manual System. Pub 100-04 Medicare claims processing. 20.4.2: Site of service payment differential. Centers for Medicare & Medicaid Services. Published 2020. Accessed January 31, 2025. https://www.cms.gov/files/document/r10356cp.pdf

[28] Robinson JC, Whaley CM, Brown TT. Price differences to insurers for infused cancer drugs in hospital outpatient departments and physician offices: study examines differences in prices insurers pay for cancer drugs in hospital outpatient clinics compare with physician offices. Health Aff. 2021;40(9):1395-1401. doi:10.1377/hlthaff.2021.00211 34495715

