# Supplementary material for: Claim Denials for Cancer-Related Next-Generation Sequencing in Medicare
Source: JAMA Netw Open. 2025 Apr 18;8(4):e255785. doi: 10.1001/jamanetworkopen.2025.5785 (PMC12008754; doi:10.1001/jamanetworkopen.2025.5785)
Supplement: Supplement 1. — eTable 1. NGS Claim Charge Rates by Testing Type and Testing Site eTable 2. Sensitivity Analysis: Adjusted Association Between NGS Claim Denial and Variables of Interest eTable 3. Reasons for Claim Denial by Testing Site eFigure. Trends in Reasons for Claim Denials for NGS Testing [file jamanetwopen-e255785-s001.pdf]

Supplementary Online Content

Kang SY, Odouard I, Gresenz CR. Claim denials for cancer-related next-generation sequencing in Medicare. *JAMA Netw Open.* 2025;8(4):e255785. doi:10.1001/jamanetworkopen.2025.5785

eTable 1. NGS Claim Charge Rates by Testing Type and Testing Site

eTable 2. Sensitivity Analysis: Adjusted Association Between NGS Claim Denial and Variables of Interest

eTable 3. Reasons for Claim Denial by Testing Site

eFigure. Trends in Reasons for Claim Denials for NGS Testing

This supplementary material has been provided by the authors to give readers additional information about their work.

© 2025 Kang SY et al. *JAMA Network Open.*

eTable 1. NGS Claim Charge Rates by Testing Type and Testing Site

Variables	Median Charge Rate (IQR)
All claims	$3,800 (2,650-3,979)
HCPCS code	
81445	$3,170 (2,370-3,979)
81450	$3,014 (2,214-4,028)
81455	$3,800 (3,800-3,988)
Site	
Hospital outpatient centers	$3,200 (2,512-4,132)
Independent laboratories	$3,800 (3,089-3,952)
Other	$3,057 (1,455-4,856)

© 2025 Kang SY et al. *JAMA Network Open.*

eTable 2. Sensitivity Analysis: Adjusted Association Between NGS Claim Denial and Variables of Interest

	Odds Ratios				
**Variable**	Model 1. Main model	Model 2. M1+ Cancer subtypes	Model 3. M1 focusing on age over 65	Model 4. M1 focusing on first NGS test	Model 5. M1 focusing on NCD 1 only
**Time period (ref. Period 1: 2016-February 2018)**					
Period 2 (March 2018- December 2019)	1.23***	1.223***	1.287***	1.245***	-
Period 3 (January 2020 - December 2021)	1.64***	1.622***	1.723***	1.839***	-
Period 2 and 3 combined (M arch 2018-December 2021)	-	-	-	-	1.467***
**Not the beneficiary's first NGS (ref: first NGS)**	1.36***	1.543***	1.346***	-	1.385***
**NGS Type (ref: 81445)**					
81450	0.41***	0.569***	0.399***	0.436***	0.416***
81455	1.32***	1.181***	1.312***	1.418***	1.411***
**Site of Testing (ref: Hospital)**					
Independent Laboratory	2.76***	3.028***	2.802***	2.445***	2.759***
Other	2.55***	2.952***	2.652***	2.420***	2.569***
**Gender (ref: Male)**					
Female	0.94	0.932*	0.947	0.899***	0.939
**Age, years (ref: 65-69)**					
<65	0.96	0.967	-	0.946	0.952
70-74	0.91*	0.924	0.904*	0.901*	0.909*
75-80	0.88**	0.946	0.883**	0.856***	0.884**
**Race / Ethnicity (ref: White)**					
Black	1.04	1.044	1.077	1.019	1.034
Asian	0.84	0.888	0.855	0.775*	0.851
Hispanic	1.29	1.227	1.192	1.123	1.289
North American Native/Unknown/ Other	1.05	1.017	1.072	1.095	1.050
**Cancer subtypes (ref: digestive organs)**					
Respiratory	-	0.372***	-	-	-
Bone, skin, or soft tissue	-	1.876***	-	-	-
Genital organs or urinary tract	-	1.737***	-	-	-
Breast	-	1.475***	-	-	-
Thyroid and endocrine glands	-	1.565***	-	-	-
Lymphoma, myeloma, or leukemia	-	0.670***	-	-	-
In situ, benign, and uncertain neoplasms	-	0.530***	-	-	-
**Observations**	29,911	29,911	27,753	24,437	29,911

© 2025 Kang SY et al. *JAMA Network Open.*

* $p < 0.05$, ** $p < 0.01$, *** $p < 0.001$
Source: 20% random sample of Medicare fee-for-service claims. State fixed effects were included in the models but not shown.

© 2025 Kang SY et al. *JAMA Network Open.*

eTable 3. Reasons for Claim Denial by Testing Site

Hospital claims (N=2075)

**Reason for denial**	**%**
This claim/service is adjusted (denied) based on the diagnosis	67.30%
These are non-covered services because this is not deemed a “medical necessity” by the payer	18.10%
Non-covered charges	5.10%
Claim denied — prior processing information appears incorrect	3.26%
Claim adjusted because this care may be covered by another payer per coordination of benefits	1.30%
Coverage/program guidelines were not met.	1.10%
Payment is included in the allowance for a Skilled Nursing Facility (SNF) qualified stay.	0.90%
The information submitted does not support this many/frequency of services.	0.70%
Missing information	2.24%
**Total**	**100.00%**

Non-institutional claims (N=4507)

**Reason for denial**	**%**
Medically unnecessary	52.36%
Other	42.96%
Non-covered care	2.35%
Multiple submittal- duplicate line item	2.29%
MIR group health plan	0.04%
**Total**	**100.00%**

© 2025 Kang SY et al. *JAMA Network Open.*

eFigure. Trends in Reasons for Claim Denials for NGS Testing

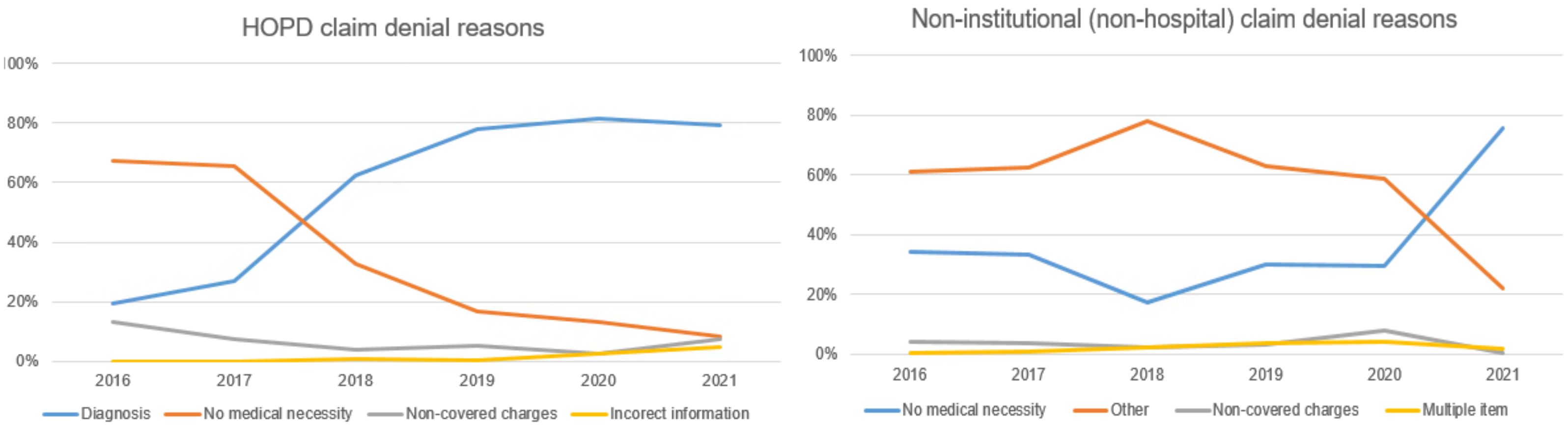

© 2025 Kang SY et al. *JAMA Network Open.*
