# Supplementary material for: Claim Denials for Cancer-Related Next-Generation Sequencing in Medicare
Source: JAMA Netw Open. 2025 Apr 18;8(4):e255785. doi: 10.1001/jamanetworkopen.2025.5785 (PMC12008754; doi:10.1001/jamanetworkopen.2025.5785)
Supplement: Supplement 2. — Data Sharing Statement [file jamanetwopen-e255785-s002.pdf]

Data Sharing Statement

Kang. Claim Denials for Cancer-Related Next-Generation Sequencing in Medicare. *JAMA Netw Open*. Published April 18, 2025. doi:10.1001/jamanetworkopen.2025.5785

Data
Data available: No
